# Finite Element Analysis Applied to Dentoalveolar Trauma: Methodology Description

**DOI:** 10.5402/2011/297132

**Published:** 2011-05-31

**Authors:** B. R. da Silva, J. J. S. Moreira Neto, F. I. da Silva, A. S. W. de Aguiar

**Affiliations:** ^1^Biotechnology Postgraduate Department, Federal University of Ceará, Sobral, CEP 62042-280, Brazil; ^2^Dentistry Department, Federal University of Ceará, Fortaleza, CE 60020-181, Brazil; ^3^Mechanical Engineering Department, Federal University of Ceará, Fortaleza, CE 60020-181, Brazil

## Abstract

Dentoalveolar traumatic injuries are among the clinical conditions most frequently treated in dental practice. However, few studies so far have addressed the biomechanical aspects of these events, probably as a result of difficulties in carrying out satisfactory experimental and clinical studies as well as the unavailability of truly scientific methodologies. The aim of this paper was to describe the use of finite element analysis applied to the biomechanical evaluation of dentoalveolar trauma. For didactic purposes, the methodological process was divided into steps that go from the creation of a geometric model to the evaluation of final results, always with a focus on methodological characteristics, advantages, and disadvantages, so as to allow the reader to customize the methodology according to specific needs. Our description shows that the finite element method can faithfully reproduce dentoalveolar trauma, provided the methodology is closely followed and thoroughly evaluated.

## 1. Introduction

Dentoalveolar trauma currently constitutes one of the main clinical conditions requiring dental treatment. Several studies have shown a high prevalence of these events, especially among children and adolescents [1–3]. 

In spite of the epidemiological importance of dentoalveolar traumatic events, little is still known about their biomechanical characteristics and their impact on adjacent tissues. Such a gap in the literature can probably be explained by the difficulties involved in the performance of sophisticated clinical and experimental studies, employing sound and reliable methodology [4, 5].

Several methodologies have been developed with the aim of improving our understanding of the distribution of forces in the stomatognathic system. Among such methodologies it is possible to mention photoelastic models, analytical mathematical models, and mathematical analyses such as the finite element method (FEM) [6].

In FEM, the behavior of a particular physical system is mathematically simulated. A continuous structure is divided into different elements, which maintain the properties of the original structure. Each of these elements is described by differential equations and solved using mathematical models selected according to the data under investigation [6–9].

FEM allows to create models for complex structures, reproducing the irregular geometries of either natural or artificial tissues, for example, the dentoalveolar articulation. In addition, FEM allows to modify the parameters of those geometries, which makes it possible to apply a force or a system of forces to any point and/or in any direction, thereby providing information on movement and on the degree of tension and compression forces caused by these loads [6, 8, 10–12].

The application of FEM to the investigation of dental trauma requires the adoption of complex methodologies. The aim of this study was to describe the methodological steps involved in the creation of a dentoalveolar articulation model using FEM for the simulation of traumatic events, with an emphasis on the characteristics and particularities of the modeling process.

## 2. Methodology Description

In order to achieve an accurate simulation of dentoalveolar trauma and obtain scientifically valid results, a minimum of six materials have to be modeled, namely, cortical bone, cancellous bone, periodontal ligament, dentin, enamel, and the dental pulp. Combining a larger number of materials definitely yields more precise results; conversely, it also poses more difficulties to the development of the model and increases the complexity of data analysis. 

For didactic purposes, the FEM model creation process was divided into five sections, namely, development of the geometric model, mesh generation, delimitation of the periodontal ligament, behavior and properties of the physical models, and analysis and evaluation of results. Each of these steps is described below.

### 2.1. Development of the Geometric Model

The first step before an FEM model can be obtained is the creation of a virtual geometry model (VGM) of the dentoalveolar articulation. The VGM will serve as the basis for the subsequent creation of the FEM, and therefore any errors at this stage may render the model and the study results irrelevant.

The type of VGM created (two- or three-dimensional) should be determined depending on the characteristics and analyses that the investigator intends to perform. Two-dimensional models are simpler, but allow anteroposterior assessments only [5, 6, 13, 14]. Three-dimensional models, in turn, are more complex but allow a more complete assessment of structures and loads, in any direction [6, 10, 12, 15, 16]. VGMs can also be described in terms of their precision, that is, models that more closely resemble the real structures will produce more reliable results [6, 12].

Basically, the VGM can be obtained in two ways: (1) by acquiring commercially available geometry models, or (2) by using a digital imaging technology, for example, computed tomography (CT) and assembling the VGM. In the first case, VGMs can be created by graphic design companies using software such as AutoCAD (Autodesk, USA) and SolidWorks (SolidWorks Corporation, USA). However, these models usually have few anatomic details and are extremely expensive [6]. The second method consists of effectively building a VGM, as described below.

**Figure 1:**
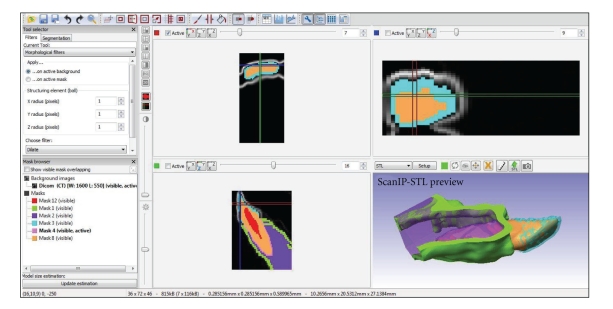
Tomographic image discretization for the creation of the virtual geometry model.

In order to create a VGM using CT, it is first necessary to collect a series of CT images (slices) of the structure to be analyzed, saved in DICOM format (Digital Imaging Communications in Medicine; NEMA, Rosslyn, USA). Next, based on the information provided by CT slices, images are exported to a software which will then reconstruct the structure on the computer (discretization process; Figure 1). Some of the software currently used for this purpose are Patran and Nastran (MSC Software, Santa Ana, USA), Mimics/MedCAD (Materialise, Leuven, Belgium), and ScanIP/ScanFE (Simpleware, Exter, UK) [15, 16]—all this software is proprietary and requires registration and a valid license. The VGM obtained as a result of CT image processing will generate a complete two- or three-dimensional image of the region or structure to be evaluated. Subsequently, the investigator can also specify the biomaterial to be studied and define specific properties, so as to produce a more reliable and suitable model [10, 17].

### 2.2. Mesh Generation

**Figure 2:**
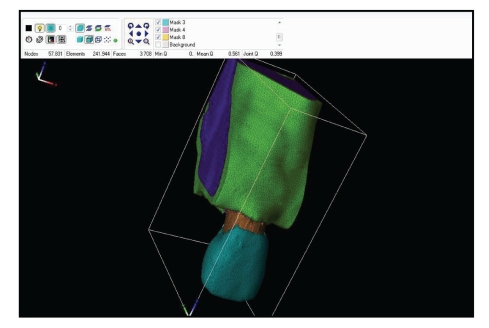
Finite element mesh of the dentoalveolar articulation.

Once the VGM of the dentoalveolar articulation has been obtained, it should be processed by another software in order to generate the finite element mesh (Figure 2).

Several software options are currently available and can be used for FEM mesh generation, with satisfactory results, particularly Ansys (Swanson Analysis Systems, Houston, PA, USA) and MSC/Nastran (MSC Software Corporation, Santa Ana, CA, USA) [5, 13, 15, 16]. These options have different interfaces and characteristics, and all are capable of generating the desired mesh and subsequently performing pre-established analyses. 

The finite element mesh comprises spatial coordinates represented by quadrilateral elements combined and arranged to produce different geometric shapes—triangles, tetrahedrons, and hexahedrons (most commonly the latter two). The higher the number of quadrilaterals used to generate the mesh, the higher the precision and reliability of FEM in relation to the VGM [6–8, 11, 12, 16].

The quadrilaterals used in mesh generation are connected by nodes, resulting in a complex two- or three-dimensional net, which allows the transport of mathematical equations between the coordinates [11, 12].

### 2.3. Delimitation of the Periodontal Ligament

Proper demarcation of the periodontal ligament is extremely important for the correct simulation of dentoalveolar trauma, especially in view of the important functions of this structure, for example, impact damping and participation in posttraumatic events [5, 18]. However, no clear instructions can be found in the literature to guide this important step; moreover, identification of the periodontal ligament on CT images is often not possible because of its small thickness.

**Figure 3:**
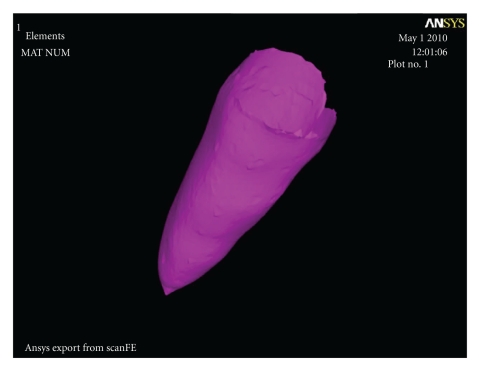
Finite element mesh of the periodontal ligament.

With the aim of faithfully demarcating this structure, a resource called surface contact method can be used [10, 15, 16, 18]. This method creates a two-dimensional layer covering all surfaces of a given structure that is in contact with another structure, previously selected. For example, when applying the contact method to the root surface of a tooth that is in contact with cancellous bone, the software creates a layer around the root surface and thus allows its configuration (Figure 3). This method makes it possible to set the thickness and other physical-mechanical properties of the periodontal ligament.

### 2.4. Behavior and Properties of Physical Models

Generally, material behavior can be classified into five categories: nonlinear elastic phenomena (return to original conditions after deformation, not following a specific pattern), plastic phenomena (deformation without return to original conditions), elastoplastic phenomena (partly elastic and partly plastic behavior), viscoelastic phenomena (return to original conditions after deformation is time dependent) and viscoplastic phenomena (time-dependent deformation without return to original conditions) [6, 7].

In the case of FEM applied to dentoalveolar trauma, it is mandatory to assign physical-mechanical properties to the structural components of the articulation on the generated mesh. This will reinforce reproducibility of the model by defining specific properties to the bone, periodontal ligament, dental pulp, dentin, and enamel.

The dentoalveolar articulation presents a viscoplastic behavior, that is, movement or deformation is dependent on the time during which the force is applied; once force application is interrupted, the tooth does not return to its original position [6].

The analysis of viscoplastic materials has some limitations, because the physical and mechanical properties of these structures are not yet completely understood. In the case of the dentoalveolar articulation, such limitations acquire increased significance when related to the periodontal ligament, the main structure involved in dentoalveolar movement and impact damping [18].

Another factor that should be taken into consideration refers to the mechanical characteristics of each material comprising the FEM. In general, materials can be classified as isotropic, anisotropic, and orthotropic. This classification is based on the mechanical properties of a material in relation to the directions of each of the axes (*X*, *Y*, and *Z*): isotropic materials are defined as those that present the same properties in every direction; in anisotropic materials, properties are different along the directions; finally, in orthotropic materials, properties are the same in two directions and different in the third [6].

In the dentoalveolar articulation structure, the dentin, dental pulp, periodontal ligament, and cortical and cancellous bone areas behave isotropically whereas tooth enamel behaves as an anisotropic material, due to the arrangement of its prisms [15, 16].

The last factors to be considered and included in the generated mesh are Poisson's ratio, Young's modulus and information on the density of each material. Each of these factors will provide the software with data on how a given material behaves when submitted to force application taking into consideration its deformation capacity, elasticity, and behavior under tension or compression (Table 1) [5, 13, 15, 16].

### 2.5. Analysis and Evaluation of Results

One of the current challenges in dentoalveolar traumatology is to improve understanding of the mechanisms underlying trauma and their impacts on dentoalveolar structures. 

Once the FEM model has been created and all its properties defined, it becomes possible to simulate the application of a given force, in Newtons (N), for a given time, in milliseconds (ms), to the structure. The literature presents variable data regarding the application of different forces for different time periods in the simulation of dentoalveolar trauma. 

According to Miura and Maeda [14], the application of a force of 100 N over a period of 1.5 ms is enough to simulate the avulsion of an upper central incisor. Other studies [5, 13, 15, 16] have indicated a force of 800 N, equally distributed over a period of 4 ms, as the correct configuration to simulate mild to moderate dentoalveolar traumatic events. This last recommendation is currently most accepted, since a load of 100 N is considered to be a normal force applied to human teeth during mastication.

Once force and time properties have been properly defined, the software performs a series of calculations and mathematical equations and yields the simulation results. These are presented according to a color scale where each shade represents a different degree of movement, tension, or compression. The model also allows to select one particular axis or structure for the analysis of tension/compression or movement, allowing simulation of a variety of traumatic events and thereby increasing the possibilities of analysis [6].

## 3. Conclusions

The methodological steps herein described indicate that FEM is a complex but effective methodology for the simulation of dentoalveolar trauma, with results that are highly precise and compatible with real clinical situations. The methodology requires thorough, specialized knowledge in the field of engineering or biomechanics, and the final analyses should be conducted with the support of professionals from these fields.

## Figures and Tables

**Table 1:** Physical-mechanical properties of the elements comprising the dentoalveolar articulation.

	Young's modulus	Density	Poisson's
	(GPa)	(g/cm^3^)	ratio
Enamel	77.90	3.00	0.33
Dentin	16.6	2.20	0.31
Pulp	0.00689	1.00	0.45
Periodontal ligament	0.05	1.10	0.45
Alveolar bone	3.50	1.40	0.33
Cortical bone	10.00	1.40	0.26
Cancellous bone	0.50	1.40	0.38
